# Granulocyte and monocyte adsorption therapy in sepsis: a propensity score-matched analysis

**DOI:** 10.1186/s40560-026-00872-9

**Published:** 2026-03-16

**Authors:** Ryo Hisamune, Kazuma Yamakawa, Tomoyuki Nakamura, Kent Doi, Gaku Takahashi, Kazuhiro Moriyama, Takuma Ishihara, Osamu Nishida

**Affiliations:** 1 Department of Emergency and Critical Care Medicine, Osaka Medical and Pharmaceutical University, 2-7 Daigakumachi, Takatsuki, Osaka, 569-8686 Japan; 2 Department of Anesthesiology and Critical Care Medicine, Fujita Health University School of Medicine, Toyoake, Aichi Japan; 3 Department of Emergency and Critical Care Medicine, The University of Tokyo, Tokyo, Japan; 4 Department of Critical Care and Emergency, Iwate Prefectural Advanced Critical Care and Emergency Center, Iwate Medical University, Shiwa, Iwate Japan; 5 Innovative and Clinical Research Promotion Center, Gifu University Hospital, Gifu, Japan

**Keywords:** Apheresis, Neutrophil, Hemoadsorption, Sepsis, Monocyte

## Abstract

**Background:**

Granulocyte and monocyte adsorption therapy has been explored as an adjunctive treatment for sepsis due to its potential to modulate excessive systemic inflammation. However, clinical evidence characterizing its real-world use for sepsis remains limited. This study aimed to conduct an exploratory comparative assessment of granulocyte and monocyte adsorption apheresis-direct hemoperfusion (G1-DHP) in patients with sepsis.

**Methods:**

We conducted a retrospective comparative study using a prospective multicenter dataset of patients treated with G1-DHP (G-1 trial) and three independent sepsis datasets (Japan Septic Disseminated Intravascular Coagulation [JSEPTIC-DIC], Focused Outcomes Research in Emergency Care in Acute Respiratory Distress Syndrome, Sepsis, and Trauma [FORECAST], and Japan Medical Data Center [JMDC]) as controls. Propensity score matching was performed using one-to-one nearest-neighbor matching. The primary outcome was 28-day mortality. Secondary outcomes included ventilator-free period, intensive care unit (ICU)-free period, and improvements in organ dysfunction scores. Ordinal logistic regression and linear regression were used based on outcome characteristics.

**Results:**

After matching, the cohorts included 71, 72, and 68 patient pairs for comparisons with JSEPTIC-DIC, FORECAST, and JMDC, respectively. 28-day mortality was significantly lower in the G-1 trial across all matched datasets (G-1 trial vs. JSEPTIC-DIC: 5.6% vs. 23%; G-1 trial vs. FORECAST: 5.6% vs. 28%; G-1 trial vs. JMDC: 5.9% vs. 38%, all *P* < 0.01). The G-1 trial had a significantly longer ventilator-free period and a trend toward a longer ICU-free period. G1-DHP was also associated with greater observed improvement in Sequential Organ Failure Assessment (SOFA) score by day 7 in comparison to controls. Improvements in liver and coagulation SOFA subscores were particularly notable.

**Conclusions:**

In this multi-dataset analysis, patients treated with G1-DHP showed lower mortality and more favorable clinical outcomes in comparison to external controls. These exploratory findings provide preliminary insights into the potential role of G1-DHP as an immunomodulatory approach in sepsis and warrant further evaluation in prospective studies.

**Supplementary Information:**

The online version contains supplementary material available at 10.1186/s40560-026-00872-9.

## Introduction

Sepsis remains a significant global health concern, characterized by high mortality rates despite advancements in critical care management [1, 2]. The Surviving Sepsis Campaign Guidelines advocate for various therapeutic strategies [3], including early administration of antibiotics, fluid resuscitation, and vasopressor support; however, a definitive treatment for sepsis remains to be established. Blood purification therapies, such as hemofiltration and adsorption techniques, have been investigated as potential adjunctive treatments aimed at modulating the excessive inflammatory response associated with sepsis [4–8]. Nevertheless, the clinical evidence supporting their efficacy remains inconclusive, and no single modality has been universally adopted in routine clinical practice.

Blood purification using a granulocyte and monocyte adsorption (GMA) column (G-1: equivalent to the commercially available Adacolumn, JIMRO Co. Ltd. Gunma) is designed to selectively remove activated granulocytes and monocytes, which play key roles in the dysregulated immune response of sepsis. Previous studies have reported its potential benefits in autoimmune diseases and inflammatory conditions, such as ulcerative colitis [9, 10]. The theoretical rationale for its use in sepsis is based on its ability to reduce excessive systemic inflammation and restore immune homeostasis. A recent multicenter single-arm clinical trial (G-1 trial) reported reductions in Sequential Organ Failure Assessment (SOFA) score before and after G-1 direct hemoperfusion (G1-DHP) treatment in patients with sepsis [11], suggesting a possible therapeutic signal. However, clinical evidence for G1-DHP in sepsis remains limited, and comparative data are lacking.

Given this context, further investigation is needed to explore the potential role of G1-DHP in sepsis management. In this study, we conducted an exploratory comparison between a single-arm G1-DHP cohort and three external large-scale control datasets to describe clinical characteristics and observed clinical outcomes. This analysis is intended to provide the preliminary clinical context for the use of G1-DHP and to indicate the need for future prospective trials.

## Materials and methods

### Study design and setting

This study is a secondary analysis using the G-1 trial, which was a prospective, multicenter, single-arm clinical trial [11]. Patients from the G-1 trial dataset, who received G1-DHP therapy for sepsis, were used as the intervention group. This dataset included 83 adult patients (age ≥ 18 years) with sepsis, as defined by Sepsis-3 criteria [12], who were admitted to 15 intensive care units (ICUs) in Japan between October 2020 and November 2022. The full inclusion and exclusion criteria for patient enrollment are summarized in Supplementary Table S1. Briefly, eligible patients were enrolled within 24 h of ICU admission, were 18–85 years of age, and had an Acute Physiologic Assessment and Chronic Health Evaluation II (APACHE II) score of 17–34. G1-DHP therapy was initiated within 3 h after enrollment. For reference, the median time from ICU admission to the start of G1-DHP was 18 h and 30 min. The G-1 trial also excluded patients who had received extracorporeal membrane oxygenation or who had a white blood cell count of < 4000 cells/mm^3^.

As controls, patients from three additional sepsis datasets were included: Japan Septic Disseminated Intravascular Coagulation (JSEPTIC-DIC) [13], Focused Outcomes Research in Emergency Care in Acute Respiratory Distress Syndrome, Sepsis, and Trauma (FORECAST) [14], and a claim dataset purchased from the Japan Medical Data Center (JMDC). The JSEPTIC-DIC study retrospectively collected data on 3195 patients (age ≥ 16 years) with sepsis who were admitted to 42 ICUs in Japan between January 2011 and December 2013. The FORECAST study included 1184 patients (age ≥ 16 years) with sepsis who were admitted to 59 ICUs in Japan between January 2016 and March 2017. The JMDC database is a large repository of health insurance claims data. It consists of data on approximately 2.77 million patients discharged between January 2019 and March 2023 from 95 hospitals with available laboratory data. The JMDC dataset contains integrated medical and pharmacy claims data, including demographics, diagnoses, examinations, drug prescriptions, and laboratory results. Diagnoses were recorded using the International Classification of Diseases, Tenth Revision (ICD-10) codes.

This study was conducted in accordance with the principles of the Declaration of Helsinki, and the study protocol was approved by the Institutional Review Board of Osaka Medical and Pharmaceutical University (Approval Number: 2025-041). Due to the anonymous nature of the data, the requirement for informed consent from patients was waived.

### Participants

We included adult patients (defined as ≥ 18 years of age) with sepsis who required intensive care from these four datasets. In JSEPTIC-DIC and FORECAST, sepsis was defined by a total SOFA score of ≥ 2 according to the Sepsis-3 definition [12]. The JMDC defined sepsis according to the ICD-10 criteria as a condition involving a combination of infection and organ dysfunction [2]. Patients who underwent G1-DHP were identified from the G-1 trial dataset [11]. No treatment limitations that could interfere with the standard treatment of sepsis were imposed on patients in the G-1 group. We excluded patients who met the following criteria: age < 18 years, receipt of extracorporeal membrane oxygenation, or missing data on APACHE II score, SOFA score, or in-hospital outcomes.

The procedure for G1-DHP followed the treatment protocol used in the G‑1 trial [11]. It was generally set at a blood flow rate of 50 mL/min for 120 min (the processed blood volume was 6000 mL), with a guideline to use five columns within 3 days. The second round commenced 12 h (± 6 h) after the first round, the third round commenced 24 h (± 6 h) after the first round, and the fourth and fifth rounds each commenced 24 h (± 6 h) after the previous use. None of the patients in the control datasets (JSEPTIC-DIC, FORECAST, and JMDC) received G1-DHP therapy.

### Data collection

We collected the following data: patient demographics (i.e., age, sex, and comorbidities), primary diagnoses on ICU admission, length of hospital stay and ICU stay, ICU treatment details (i.e., mechanical ventilation, renal replacement therapy, vasopressor agents, and low-dose steroids), and in-hospital mortality. Comorbidities were defined using the Charlson comorbidity index. In the JMDC dataset, details on the definition of the ICD-10 codes used in this study are provided in Supplementary Table S2.

Clinical data on the APACHE II score, SOFA score, disseminated intravascular coagulation (DIC) score, and laboratory results were recorded at baseline, day 3, and day 7 after ICU admission. Laboratory tests included platelet count, prothrombin time-international normalized ratio, fibrinogen level, fibrin/fibrinogen degradation products, D-dimer, and white blood cell count. The DIC score was assessed using the Japanese Association for Acute Medicine (JAAM)-2 DIC criteria. Due to the specific characteristics of each dataset, there were differences in the available outcome data. The FORECAST dataset included clinical scores and laboratory data only up to day 3 after ICU admission. In the JMDC dataset, only SOFA subscores for liver, renal, and coagulation functions derived from laboratory data were available, and APACHE II scores were not included.

### Definition of outcomes

The primary outcome was 28-day mortality. Secondary outcomes included the 28-day ventilator-free period, 28-day ICU-free period, and changes in the SOFA score and DIC score from day 1 to days 3 and 7. The ventilator-free and ICU-free periods were assigned a value of zero for patients who died within 28 days. Due to the characteristics of each dataset, the comparison between the G-1 and FORECAST studies focused on changes in SOFA and DIC scores from day 1 to day 3, whereas the comparison between the G-1 and JMDC studies was limited to SOFA subscores (liver, kidney, and coagulation).

### Statistical analysis

Due to the retrospective nature of the study, an imbalance among covariates was assumed across the datasets. Therefore, propensity score matching was performed between patients in the G-1 dataset and those in the other datasets. Propensity scores were calculated using a logistic regression model based on dataset-specific variables (Supplementary Table S3). In the step of estimating propensity scores, missing values were imputed using additive regression. A one-to-one nearest-neighbor matching approach without replacement was applied, with a caliper of SD × 0.2 of the estimated log odds value. Standardized mean differences (SMDs) were calculated to assess balance before and after matching, with values < 0.1 considered acceptable. Variables with SMD ≥ 0.1 were included as covariates in the analysis to reduce potential bias. We assessed 28-day survival using Kaplan–Meier survival curves and the log-rank test. To estimate risk ratios, we used modified Poisson regression models with a log link function. In addition, risk differences were evaluated using modified Poisson regression models with an identity link function. Additionally, we assessed secondary outcomes using regression models appropriate to the nature of each outcome variable. Ordinal logistic regression (using the lrm() function from the rms package in R) was applied to non-normally distributed ordinal outcomes, such as the ICU-free and ventilator-free periods. Continuous outcomes with approximately normal distributions, such as SOFA and DIC scores, were analyzed using ordinary least squares regression (using the ols() function from the rms package in R). As a sensitivity analysis, an additional model was fitted including variables with SMD ≥ 0.1 after matching as covariates. In addition, inverse probability of treatment weighting (IPTW) was performed to estimate the average treatment effect and to further assess the robustness of the treatment effect estimates.

Descriptive statistics are calculated as medians (interquartile range) or proportions (number), as appropriate. Univariate differences between groups were assessed using the Mann–Whitney U test or Chi-square test, as appropriate. P values of < 0.05 were considered to indicate statistical significance. Data were analyzed using R (ver. 4.2.3, The R Foundation for Statistical Computing, Vienna, Austria).

## Results

### Study population

**Fig. 1:**
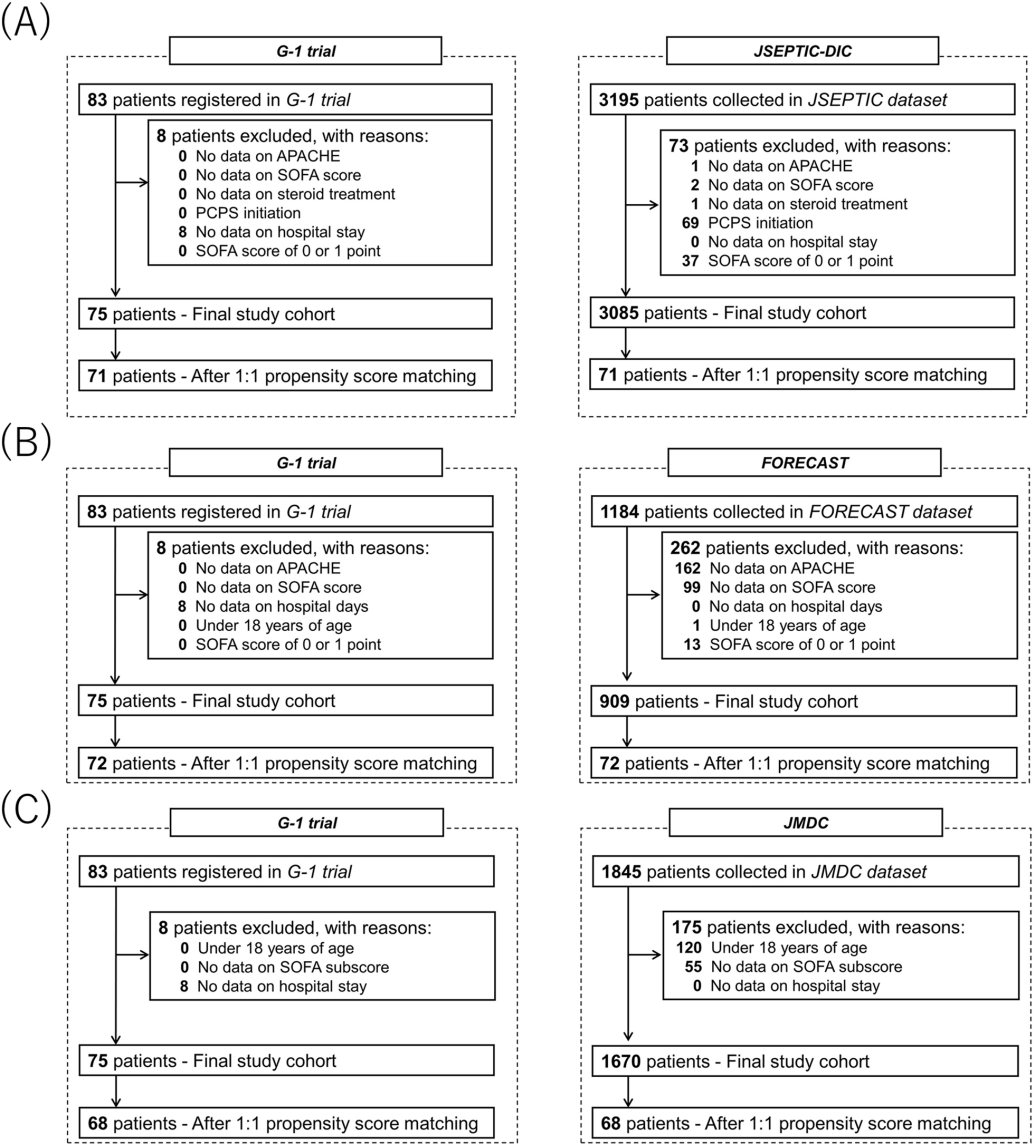
Flowchart of patient selection for each comparison. **A** G-1 vs. JSEPTIC-DIC. **B** G-1 vs. FORECAST. **C** G-1 vs. JMDC. *SOFA* Sequential Organ Failure Assessment, *APACHE* Acute Physiologic Assessment and Chronic Health Evaluation, *PCPS* percutaneous cardiopulmonary support, *JSEPTIC-DIC* Japan Septic Disseminated Intravascular Coagulation, *FORECAST* Focused Outcomes Research in Emergency Care in Acute Respiratory Distress Syndrome, Sepsis, and Trauma, *JMDC* Japan Medical Data Center

We identified 83 patients in the G-1 trial, 3195 in JSEPTIC-DIC, 1184 in FORECAST, and 1845 in JMDC. After applying the exclusion criteria, 75, 3085, 909, and 1670 patients, respectively, were eligible for the analysis (Table 1). In the final analysis, patients in the G-1 trial were matched separately with those in each of the other datasets. The matched cohorts consisted of 71 patients in the G-1 trial and 71 in JSEPTIC-DIC, 72 in the G-1 trial and 72 in FORECAST, and 68 in the G-1 trial and 68 in JMDC (Fig. 1). Supplementary Tables S4-6 show the baseline characteristics after propensity score matching. The mean age was approximately 68 years. The severity of illness was indicated by a median SOFA score of 9. Supplementary Tables S4-6 also show the therapies provided in the ICU and the laboratory test results on the day of ICU admission after propensity score matching. Regarding ICU management, approximately 70% of the patients received mechanical ventilation, approximately 90% received vasopressor medications, and approximately 80% underwent renal replacement therapy.

**Table 1:** Patient characteristics in each dataset

	G-1 trial	JSEPTIC-DIC	FORECAST	JMDC
Study period	2020.10–2022.11	2011.11–2013.12	2016.1–2017.3	2019.1–2023.3
Number of patients	75	3085	909	1670
Age, years	68.81 (11.34)	69.88 (14.36)	70.30 (14.61)	74.39 (14.10)
Sex, male	41 (54.7)	1845 (59.8)	565 (62.2)	1014 (60.7)
APACHE II	26.59 (4.43)	23.22 (8.67)	23.74 (8.84)	–
Total SOFA score	9.71 (3.11)	9.40 (4.02)	8.76 (3.73)	–
*SOFA subscores*				
Liver function	0.71 (0.93)	–	–	0.44 (0.77)
Kidney function	1.72 (1.33)	–	–	1.24 (1.34)
Coagulation function	1.24 (1.18)	–	–	0.70 (0.97)
*Past history*				
Heart failure	7 (9.3)	175 (5.7)	143 (15.7)	595 (35.6)
Chronic kidney disease	10 (13.3)	254 (8.2)	66 (7.3)	309 (18.5)
Chronic pulmonary disease	5 (6.7)	119 (3.9)	62 (6.8)	253 (15.1)
Chronic liver disease	0 (0)	127 (4.1)	51 (5.6)	192 (11.5)
*Infection site*				
Urinary tract	21 (28.0)	505 (16.4)	158 (17.4)	129 (7.7)
Lung	12 (16.0)	792 (25.7)	294 (32.1)	433 (25.9)
Intra-abdominal	26 (34.7)	1004 (32.5)	243 (26.7)	411 (24.6)
Other	12 (16.0)	572 (18.5)	164 (18.0)	117 (7.0)
No data	4 (5.3)	212 (6.9)	52 (5.7)	580 (34.7)
*Causative microorganism*				
Gram-negative rod	19 (25.3)	1138 (36.9)	326 (35.9)	–
Gram-positive coccus	13 (17.3)	710 (23.0)	249 (27.4)	–
Fungus	1 (1.3)	53 (1.7)	14 (1.5)	–
Mixed	41 (54.7)	378 (12.3)	97 (10.7)	–
Other	1 (1.3)	806 (26.1)	223 (24.5)	–
*Intensive care treatment*				
Mechanical ventilation	53 (70.7)	2209 (71.6)	406 (44.7)	829 (49.6)
Vasopressor medication	67 (89.3)	2413 (78.2)	640 (70.4)	1190 (71.3)
Renal replacement therapy	61 (81.3)	1097 (35.6)	293 (32.2)	333 (19.9)
PMX	4 (5.3)	667 (21.6)	81 (8.9)	22 (1.3)
Low-dose steroid medication	49 (65.3)	739 (24.0)	289 (31.8)	513 (30.7)
*Laboratory results on the day of ICU admission*				
White blood cell (× 10^3^/μL)	16.11 (9.70)	12.92 (12.35)	13.17 (13.42)	12.86 (9.23)
Platelet counts (× 10^3^/μL)	145.17 (126.41)	143.35 (106.21)	170.91 (150.48)	187.62 (109.25)
Hemoglobin (g/dL)	10.47 (1.89)	10.78 (2.53)	11.54 (2.87)	11.56 (2.58)
Fibrinogen (mg/dL)	404.39 (143.27)	421.48 (218.39)	470.21 (222.97)	422.07 (215.98)
PT-INR	1.51 (0.64)	1.55 (1.12)	1.41 (0.78)	1.42 (0.83)
FDP (μg/mL)	43.69 (57.84)	46.02 (108.29)	43.73 (89.10)	41.11 (82.69)
D-dimer (μg/mL)	17.78 (18.80)	23.84 (171.97)	19.22 (50.78)	17.44 (35.82)
Lactate (mmol/L)	3.68 (3.33)	4.40 (4.16)	4.11 (3.47)	-
DIC score (mean (SD))	4.13 (2.11)	4.07 (2.24)	3.83 (2.16)	2.45 (1.99)

Continuous variables are presented as means with standard deviation, and categorical variables are presented as numbers with percentages

*SD* standard deviation, *SOFA* Sequential Organ Failure Assessment, *APACHE* Acute Physiologic Assessment and Chronic Health Evaluation, *JSEPTIC* Japan Septic Disseminated Intravascular Coagulation, *FORECAST* Focused Outcomes Research in Emergency Care in Acute Respiratory Distress Syndrome, Sepsis, and Trauma, *JMDC* Japan Medical Data Center, *PMX* polymyxin B-immobilized fiber, *ICU* intensive care unit, *PT-INR* prothrombin time-international normalized ratio, *FDP* Fibrin/fibrinogen degradation products

### Comparison of 28-day mortality

**Fig. 2:**
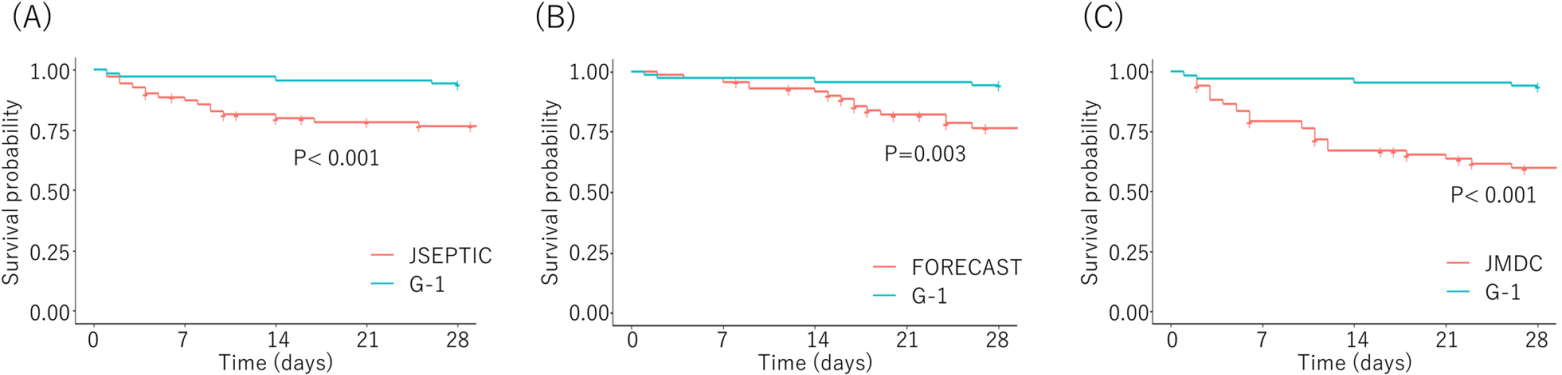
Kaplan–Meier survival curve for each propensity score-matched cohort. **A** G-1 vs. JSEPTIC-DIC. **B** G-1 vs. FORECAST. **C** G-1 vs. *JMDC JSEPTIC-DIC* Japan Septic Disseminated Intravascular Coagulation, *FORECAST* Focused Outcomes Research in Emergency Care in Acute Respiratory Distress Syndrome, Sepsis, and Trauma, *JMDC* Japan Medical Data Center

After propensity score matching, the SMDs of the covariates indicated better balance in each matched dataset (Supplementary Fig. 1). The 28-day mortality rate was lower in the G-1 trial across all matched cohorts. Unadjusted risk ratios (RRs) and risk differences (RDs) confirmed this trend (RR: JSEPTIC-DIC, 0.25 [95% CI 0.09–0.71]; FORECAST, 0.20 [0.07–0.56]; JMDC, 0.15 [0.06–0.42]; RD: JSEPTIC-DIC,−0.17 [95% CI −0.28–−0.06]; FORECAST, −0.22 [−0.34–−0.11]; JMDC, −0.32 [−0.45–−0.20]) (Table 2, Supplementary Table S7). Consistent findings were observed in the survival analysis, as shown in the Kaplan–Meier curves (Fig. 2).

**Table 2.** Unadjusted risk ratios for primary outcomes after propensity-matching

Reference	G-1 trial	Control	RR	95% LCI	95% UCI	*P*-value
JSEPTIC-DIC	4/71 (5.6%)	16/71 (22.5%)	0.25	0.09	0.71	0.009
FORECAST	4/72 (5.6%)	20/72 (27.8%)	0.20	0.07	0.56	0.002
JMDC	4/68 (5.9%)	26/68 (38.2%)	0.15	0.06	0.42	<0.001

Categorical variables are presented as numbers with percentages

*RR* risk ratio, *LCI* low confidence interval, *UCI* upper confidence interval, *JSEPTIC-DIC* Japan Septic Disseminated Intravascular Coagulation, *FORECAST* Focused Outcomes Research in Emergency Care in Acute Respiratory Distress Syndrome, Sepsis, and Trauma, *JMDC* Japan Medical Data Center

### Comparison of ICU treatment-related outcomes

Table 3 shows the secondary outcomes after propensity score matching. The ICU-free period was significantly longer in the G-1 trial in comparison to the JSEPTIC-DIC data (G-1 vs. JSEPTIC-DIC: 19 days vs. 15 days, *P* = 0.048). The ICU-free period also tended to be longer in the G-1 trial in comparison to the FORECAST and JMDC data, although the differences were not statistically significant (G-1 vs. FORECAST: 20 days vs. 17 days, *P* = 0.08; G-1 vs. JMDC: 20 days vs. 15 days, *P* = 0.30). The ventilator-free period was significantly longer in the G-1 trial across all matched datasets (G-1 vs. JSEPTIC-DIC: 24 days vs. 19 days, *P* < 0.01; G-1 vs. FORECAST: 25 days vs. 16 days, *P* < 0.01; G-1 vs. JMDC: 24 days vs. 18 days, *P* < 0.01).

**Table 3.** Secondary outcomes related to medical resource use after propensity score matching

Outcome reference	G-1 trial	Control	Unadjusted OR	95% LCI	95% UCI
*ICU free periods (days*)					
JSEPTIC-DIC	19 [12, 21]	15 [0, 21]	1.81	1.01	3.26
FORECAST	20 [13, 21]	17 [8, 21]	1.73	0.93	3.22
JMDC	20 [11, 21]	15 [0, 22]	1.37	0.76	2.50
*Ventilator free periods (days)*					
JSEPTIC-DIC	24 [19, 28]	19 [0, 28]	2.46	1.35	4.47
FORECAST	25 [20, 28]	16 [0, 22]	5.68	3.04	10.61
JMDC	24 [19, 28]	18 [1, 27]	2.59	1.41	4.77

Continuous variables are presented as median with IQR

*OR* odds ratio, *LCI* low confidence interval, *UCI* upper confidence interval, *ICU* intensive care units, *JSEPTIC-DIC* Japan Septic Disseminated Intravascular Coagulation, *FORECAST* Focused Outcomes Research in Emergency Care in Acute Respiratory Distress Syndrome, Sepsis, and Trauma, *JMDC* Japan Medical Data Center

### Changes in organ dysfunction scores

The changes in SOFA and DIC scores from day 1 to day 3 (ΔSOFA [day 3–day 1], ΔDIC [day 3–day 1]) were comparable between groups in the G-1 trial vs. JSEPTIC-DIC (ΔSOFA −0.41 vs. 0.67, *P* = 0.11; ΔDIC −0.16 vs. −0.30, *P* = 0.69) and the G-1 trial vs. FORECAST comparisons (ΔSOFA −0.59 vs. 1.11, *P* = 0.03; ΔDIC−0.30 vs. −0.07, *P* = 0.56). In contrast, the improvement in ΔSOFA (day 7–day 1) in the G-1 trial was significantly greater in comparison to the JSEPTIC-DIC (−4.40 vs. −1.83, *P* < 0.01). In the comparison between the G-1 trial vs. JMDC, although the changes in SOFA subscores from day 1 to day 3 were comparable, the improvements in liver and coagulation subscores from day 1 to day 7 were significantly greater in the G-1 trial (G-1 vs. JMDC: liver −0.08 vs. 0.31, *P* = 0.05; coagulation −0.14 vs. 0.43, *P* = 0.02) (Table 4).

**Table 4.** Secondary outcomes related to organ dysfunction after propensity score matching

	G-1 trial	Control	Coefficient	95% LCI	95% UCI	*P*-value
ΔSOFA score						
*ΔSOFA (day7 – day1)*						
JSEPTIC-DIC	−4.40 (3.88)	−1.83 (4.45)	−2.567	−4.029	−1.104	0.001
*ΔSOFA (day3 – day1)*						
JSEPTIC-DIC	−0.41 (4.09)	0.67 (3.66)	−1.072	−2.397	0.252	0.112
FORECAST	−0.59 (4.23)	1.11 (4.78)	−1.697	−3.241	−0.152	0.032
*ΔSOFA liver score (day7 – day1)*						
JMDC	−0.08 (0.85)	0.31 (1.10)	−0.385	−0.768	−0.001	0.049
*ΔSOFA kidney score (day7 – day1)*						
JMDC	−0.54 (1.52)	−0.40 (1.46)	−0.138	−0.736	0.459	0.647
*ΔSOFA coagulation score (day7 – day1)*						
JMDC	−0.14 (1.17)	0.43 (1.26)	−0.566	−1.046	−0.085	0.022
*ΔSOFA liver score (day3 – day1)*						
JMDC	0.06 (0.93)	0.29 (0.85)	−0.228	−0.544	0.089	0.157
*ΔSOFA kidney score (day3 – day1)*						
JMDC	0.35 (1.67)	0.16 (1.13)	0.187	−0.299	0.672	0.448
*ΔSOFA coagulation score (day3 – day1)*						
JMDC	0.86(1.07)	0.73 (0.80)	0.130	−0.204	0.465	0.443
ΔDIC score						
*ΔDIC score (day7 – day1)*						
JSEPTIC-DIC	−1.47 (2.40)	−1.25 (2.30)	−0.216	−1.045	0.612	0.606
JMDC	−1.42 (2.37)	0.71 (2.62)	−2.121	−3.434	−0.808	0.002
*ΔDIC score (day3 – day1)*						
JSEPTIC-DIC	−0.16 (2.10)	−0.30 (2.12)	0.144	−0.574	0.862	0.693
FORECAST	−0.30 (2.01)	−0.07 (2.33)	−0.229	−0.994	0.537	0.555
JMDC	−0.14 (2.07)	0.83 (2.41)	−0.962	−2.003	0.079	0.070

Continuous variables are presented as means with standard deviation

*LCI* low confidence interval, *UCI* upper confidence interval, *SOFA* Sequential Organ Failure Assessment, *DIC* disseminated intravascular coagulation, *JSEPTIC-DIC* Japan Septic Disseminated Intravascular Coagulation, *FORECAST* Focused Outcomes Research in Emergency Care in Acute Respiratory Distress Syndrome, Sepsis, and Trauma, *JMDC* Japan Medical Data Center

### Sensitivity analysis

To evaluate the robustness of the primary findings, a sensitivity analysis was conducted by adjusting for covariates with SMDs > 0.1 after propensity score matching. After adjustment for residual imbalances, G1-DHP remained significantly associated with reduced 28-day mortality across all cohorts (adjusted RR: JSEPTIC-DIC, 0.25 [95% CI 0.09–0.71]; FORECAST, 0.17 [0.06–0.47]; JMDC, 0.20 [0.07–0.55]), consistent with unadjusted estimates (Supplementary Table S8). Similar associations were observed using alternative estimators, including risk differences and hazard ratios (Supplementary Tables S9 and S10). In a Cox proportional hazards model with IPTW, the estimated treatment effect was consistent with the results of the propensity score-matched analysis (Supplementary Table S11). Consistent results were also observed for secondary outcomes, including ICU length of stay, ventilator-free days, improvement in SOFA scores, and resolution of DIC, further supporting the robustness of the treatment effect (Supplementary Tables S12 and S13).

## Discussion

### Principal findings

In this exploratory propensity score-matched analysis, the G1-DHP cohort showed lower observed 28-day mortality than the matched external control groups across all three datasets. The G1-DHP group also exhibited longer ventilator-free and ICU-free periods. Differences in organ dysfunction trajectories were modest, although greater improvement in SOFA score by day 7 was observed in the G1-DHP cohort in some comparisons. These findings characterize the clinical profile and observed outcomes of patients treated with G1-DHP and may help inform future investigations, including randomized controlled trials.

### Granulocyte and monocyte adsorption therapy application in sepsis

Granulocyte and monocyte adsorption therapy, a blood purification technique designed to remove activated granulocyte and monocyte by adsorption, was originally developed to treat chronic autoimmune and inflammatory diseases, including ulcerative colitis [15, 16]. In these conditions, the selective removal of these activated immune cells has been shown to reduce systemic inflammation and restore immune homeostasis [17, 18]. While ulcerative colitis is marked by localized inflammation, sepsis is characterized by a far more acute and aggressive systemic inflammatory response, often leading to rapid organ dysfunction [19]. The pathophysiology of sepsis involves complex interactions among immune cells, platelets, endothelial cells, and the complement system, resulting in a dysregulated host response [19]. Among these components, activated neutrophils play a central role in initiating the hyperinflammatory phase [11, 20]. Once activated, neutrophils release a cascade of mediators including pro-inflammatory cytokines that, while essential for host immune defense, can also damage host tissues [21–23]. Ex vivo studies [24] in patients with COVID-19 have shown that GMA therapy preferentially adsorbed activated neutrophils expressing cluster of differentiation (CD) 11b and CD66b, as well as immature subsets characterized by low CD11c expression levels [25]. These cell types are believed to play key roles in early-phase inflammatory injury. Moreover, several studies have shown that GMA therapy reduces circulating levels of pro-inflammatory mediators, such as IL-6, IL-8, and TNF-α [26–28]. Given this mechanism, GMA therapy is particularly attractive in the early phase of sepsis. G1-DHP employs a GMA column as a “decoy column” to selectively adsorb activated neutrophils and monocytes. By capturing these cells within the column, their activity in the systemic circulation is suppressed, thereby modulating the excessive inflammatory response of sepsis.

### Impact of G1-DHP on mortality and organ dysfunction

In this study, patients who received G1-DHP tended to show greater improvement in SOFA scores by day 7 in comparison to the matched controls, although both groups demonstrated a baseline decrease of approximately 2 points. Previous studies have suggested that a reduction of at least two points in the SOFA score is associated with improved survival in patients with sepsis [29, 30]; however, the extent to which these observed differences can be attributed to G1‑DHP remains unclear, given the structural differences between cohorts.

Although our study did not demonstrate a clear difference in SOFA lung subscores, the G1-DHP cohort had a longer ventilator-free period in comparison to the controls. This trend is consistent with findings from experimental sepsis models [31, 32], where GMA was associated with reduced inflammatory cell infiltration in the lungs, decreased cytokine levels in bronchoalveolar lavage fluid, and improved histopathological lung injury scores. However, the relevance of these experimental findings to clinical outcomes in sepsis remains uncertain. Further prospective studies are needed to clarify whether G1-DHP contributes to pulmonary recovery or earlier liberation from mechanical ventilation.

Regarding the DIC score, improvement was generally greater in the G-1 trial than in the controls, with the difference most pronounced on day 7. In sepsis, activated neutrophils adhere to the vascular endothelium, causing endothelial damage and increased vascular permeability. These changes contribute to the development of multiple organ failure [33]. Moreover, downregulation of the CXCR2 expression on neutrophils impairs their migration to sites of infection [34], prolongs their lifespan [35], and causes their accumulation in the circulation [21], thereby promoting uncontrolled inflammation and subsequent organ injury. G-1 can adsorb neutrophils independently of the expression of CXCR2 [36], potentially helping to modulate these neutrophil dynamics in septic endothelial injury. While these mechanistic considerations offer a biological rationale for the observed differences, they do not establish causality. Additional studies are required to better characterize the functional roles of adsorbed cells and their potential clinical implications.

### Considerations for interpretation in relation to the study design

The observed differences in this study should be interpreted with caution. The G‑1 trial dataset applied strict inclusion and exclusion criteria, including the exclusion of patients expected to die within 3 days. Consequently, the mortality rate in the G‑1 cohort was substantially lower than would typically be expected for sepsis patients with similar SOFA scores. These selection criteria cannot be reproduced in the external datasets, leading to inherent structural non-comparability between cohorts.

Although propensity score matching balanced measured covariates to the extent feasible, unmeasured confounding related to patient selection, illness trajectory, and clinical decision-making could not be fully addressed. Therefore, the observed differences in survival and organ dysfunction should be interpreted within the context of these design constraints rather than as evidence of treatment efficacy.

Nevertheless, as GMA therapy is not yet widely available in clinical practice, external comparators for early exploratory evaluation are limited. This study provides descriptive insights into the clinical course of patients treated with G1‑DHP and offers preliminary information that may help guide the design of future randomized controlled trials to rigorously assess its therapeutic potential.

### Limitations

This study has several limitations. First, it combined prospective and retrospective datasets, leading to heterogeneity in data completeness and quality that may have introduced bias. Treatment decisions were made at the discretion of physicians, and detailed timing of antibiotic agents and adjunctive therapies after the diagnosis of sepsis, including G1-DHP initiation, was unavailable. Second, this study utilized multiple datasets collected over different time periods, and temporal changes in sepsis management may have influenced the observed outcomes. Although the JMDC dataset, which reflects real-world clinical data from the same period as the G-1 trial, showed similar results, residual temporal confounding cannot be excluded. Third, differences in available baseline variables and covariates across external control datasets required the construction of separate propensity score models, which may have introduced residual confounding. Only mortality and ventilator-free days were consistently available across all datasets. Therefore, these outcomes provide the most reliable basis for interpretation. Other secondary outcomes, such as SOFA and DIC scores, were not uniformly available and should be interpreted as exploratory. Furthermore, analyses of day 7 SOFA and DIC scores were limited to patients who survived to day 7, introducing potential survivor bias. Finally, the external datasets differed in data completeness, institutional practices, and patient management strategies. These factors may have contributed to differences in observed outcomes. Even with these limitations, the primary findings were consistent across multiple sensitivity analyses, supporting the overall robustness of the findings. Future studies should ideally include contemporaneous control groups to minimize temporal heterogeneity and strengthen causal inference.

## Conclusions

In this retrospective analysis using multiple datasets, we conducted the first comparative assessment of patients treated with G1-DHP for sepsis. Across datasets, the G1-DHP cohort showed lower mortality and more favorable trajectories in organ dysfunction measures in comparison to external controls. These findings provide descriptive insights into the clinical course of patients receiving G1-DHP and suggest that further investigation into its immunomodulatory mechanism may be warranted. Given the heterogeneity across datasets and the inherent limitations of external comparative studies, these findings should be interpreted as exploratory. Further validation through prospective studies with contemporaneous control groups is needed.

## Supplementary Information

Supplementary Material 1.

## Data Availability

The datasets generated and/or analyzed during the current study were derived from four anonymized databases. Due to privacy and data use restrictions, these datasets are not publicly available, but they are available from the corresponding author on reasonable request.

## Abbreviations

GMA: Granulocyte and monocyte adsorption
G1-DHP: G-1 direct hemoperfusion
SOFA: Sequential Organ Failure Assessment
ICU: Intensive care unit
JSEPTIC-DIC: Japan Septic Disseminated Intravascular Coagulation
FORECAST: Focused Outcomes Research in Emergency Care in Acute Respiratory Distress syndrome, Sepsis, and Trauma
JMDC: Japan Medical Data Center
ICD-10: International Classification of Diseases, Tenth revision
APACHE II: Acute Physiologic Assessment and Chronic Health Evaluation II
DIC: Disseminated intravascular coagulation
JAAM: Japanese Association for Acute Medicine
IPTW: Inverse probability of treatment weighting
