# Supplementary material for: Granulocyte and monocyte adsorption therapy in sepsis: a propensity score-matched analysis
Source: J Intensive Care. 2026 Mar 16;14:39. doi: 10.1186/s40560-026-00872-9 (PMC13104446; doi:10.1186/s40560-026-00872-9)

Supplemental materials

Granulocyte and Monocyte Adsorption Therapy in Sepsis: A Propensity Score-Matched Analysis

Ryo Hisamune[1], Kazuma Yamakawa[1], Tomoyuki Nakamura[2], Kent Doi[3], Gaku Takahashi[4], Kazuhiro Moriyama[2], Takuma Ishihara[5] and Osamu Nishida[2]

[1]Department of Emergency and Critical Care Medicine, Osaka Medical and Pharmaceutical University, Takatsuki, Osaka, Japan

[2]Department of Anesthesiology and Critical Care Medicine, Fujita Health University School of Medicine, Toyoake, Aichi, Japan.

[3]Department of Emergency and Critical Care Medicine, The University of Tokyo, Tokyo, Japan.

[4]Department of Critical Care and Emergency, Iwate Prefectural Advanced Critical Care and Emergency Center, Iwate Medical University, Shiwa, Iwate, Japan

[5]Innovative and Clinical Research Promotion Center, Gifu University Hospital, Gifu, Gifu, Japan.

*__Corresponding author:__ Kazuma Yamakawa, MD, PhD

Department of Emergency and Critical Care Medicine, Osaka Medical and Pharmaceutical University, 2-7 Daigakumachi, Takatsuki, Osaka 569-8686, Japan.

Office: +81-72-683-1221; Fax: +81-72-684-6523

E-mail: kazuma.yamakawa@ompu.ac.jp

Supplementary Table S1. The full inclusion and exclusion criteria in G-1 dataset

Category	Subcategory of variables
Inclusion criteria	1) diagnosed with sepsis according to the Sepsis-3 definition
	2) aged between 18 and 85 years
	3) APACHE II score of 17–34
Exclusion criteria	1) patients predicted to die within 3 days
	2) patients who had not undergone surgical procedures for removal of infection foci
	3) patients who had an organ transplantation within the previous year
	4) patients suspected of having human immunodeficiency virus or human T-lymphotropic virus type I infection
	5) patients receiving long-term treatment for immunodeficiency
	6) patients determined to have an increased tendency to hemorrhage due to the use of G-1
	7) patients with a white blood cell count < 4000 cells/mm^3
	8) patients who had received extracorporeal membrane oxygenation

APACHE, acute physiologic assessment and chronic health evaluation

Supplementary Table S2. Definition of the presence of past history and sepsis diagnosis

Group	Code type	ICD-10 code
Past history		
Heart failure	ICD 10 codes in the concomitant or complication diagnoses	I09.9, I11.0, I13.0, I13.2, I25.5, I42.0, I42.5–I42.9, I43.x, I50.x, P29.0
Chronic renal disease	ICD 10 codes in the concomitant or complication diagnoses	I12.0, I13.1, N03.2–N03.7, N05.2–N05.7, N18.x, N19.x, N25.0, Z49.0–Z49.2, Z94.0, Z99.2
Chronic pulmonary disease	ICD 10 codes in the concomitant or complication diagnoses	I27.8, I27.9, J40.x–J47.x, J60.x–J67.x, J68.4, J70.1, J70.3
Chronic liver disease	ICD 10 codes in the concomitant or complication diagnoses	B18.x, K70.0–K70.3, K70.9, K71.3–K71.5, K71.7, K73.x, K74.x, K76.0, K76.2–K76.4, K76.8, K76.9, Z94.4, I85.0, I85.9, I86.4, I98.2, K70.4, K71.1, K72.1, K72.9, K76.5, K76.6, K76.7
Sepsis diagnosis		
Explicit sepsis	ICD-10 codes in the primary diagnosis	A021, A207-A209, A217-A219, A227-A229, A241-A244, A267, A269, A282-A289, A327-A329, A390, A394-A399, A40-A41, A427-A429, A50, A5486, B007-B009, B377-B379, N980, O030, O035, O045, O080, O23-O23.93, O41.1-O41.93, O75.3, O85-O86.89, O883, O91, O98, O982-O989, P002, P22-P23, P35-P39, R652
Implicit sepsis	ICD-10 codes in the primary diagnosis	A01, A020, A03-A09, A19, A200-A203, A210-A213, A220-A222, A23, A240, A25, A260, A27, A280-A281, A31, A320-A321, A36A38, A391-A393, A420-A422, A43-A46, A48-A49, A59, A65, A690-A691, A74-A75, A77-A79, A80-A81, A83-A89, A90-A96, A98-A99, B000-B005, B01-B09, B25-B27, B33-B34, B370-B376, B38-B50, B54-B55, B58-B64, B67, B91, B95-B99, G00-G08, G14, H050, H602, H700, I00, I02, I26, I33, I38-I39, I40, I76, I96, I981, J01-J06, J09-J22, J36, J390-J391, J85-J86,

		K35- K37, K57, K61, K630-K631, K65, K678, K750- K751, K753, K763, K770, K810, K812, K830, K9501, K9581, L02-L08, M00-M02, M86, M896, N10, N151-N159, N30, N390, N410, N412-N413, N45, N70-N77, T802, T814, T826, T827, T835, T836, T845-T847, T857, T880, U04
Organ dysfunction	ICD-10 codes in the primary diagnosis	D65, D695, E872-E878, G934, I46, I951-I959, J80, J952-J953, J96, K72, N00-N17, R090, R092, R40, R418, R55, R57
Infection site		
Lung	ICD-10 codes in the primary diagnosis	A15, A16, A31, A37, B371, B440, B441, B450, J01-J06, J13-J18, J20-J22 J440, J441, J47, J69, J85, J86
Intra-abdominal	ICD-10 codes in the primary diagnosis	A020, A04, A05, A08, A09, A183, K35-K37, K57, K61, K630, K631, K65, K750, K751, K800, K801, K803, K804, K808, K810, K830, K918
Urinary tract	ICD-10 codes in the primary diagnosis	N10- N12, N151, N159, N160, N30, N34, N390, N41, N74, N75, T835, T836
No data	ICD-10 codes in the primary diagnosis	A021, A207-A209, A217-A219, A227-A229, A241-A244, A267, A269, A282-A289, A327-A329, A390, A394-A399, A40-A41, A427-A429, A50, A5486, B007-B009, B377-B379, N980, O030, O035, O045, O080, O23-O23.93, O41.1-O41.93, O75.3, O85-O86.89, O883, O91, O98, O982-O989, P002, P22-P23, P35-P39, R652
Others	ICD-10 codes in the primary diagnosis	None of above

ICD; International Classification of Diseases

Supplementary Table S3. The 17 variables used to calculate propensity scores in the regression model

Category of variables	Subcategory of variables
Patient characteristics	1) age, 2) sex
Disease severity	3) APACHE II (JSEPTIC-DIC and FORECAST), 4) SOFA score (JSEPTIC-DIC and FORECAST: total score, JMDC: subscore)
Pre-existing condition	5) heart failure, 6) chronic heart failure, 7) chronic liver disease
Laboratory results on admission	8) platelet counts, 9) D-dimer, 10) FDP, 11) PT-INR
Intensive treatment	12) mechanical ventilation, 13) renal replacement therapy, 14) vasopressor medication, 15) low dose steroid, 16) PMX
Infection site	17) Infection site

APACHE, acute physiologic assessment and chronic health evaluation; JSEPTIC-DIC, Japan Septic Disseminated Intravascular Coagulation; FORECAST, Focused Outcomes Research in Emergency Care in Acute Respiratory Distress Syndrome, Sepsis, and Trauma; SOFA, sequential organ failure assessment score; FDP, Fibrin/fibrinogen degradation products; PT-INR, prothrombin time-international normalized ratio; PMX, polymyxin B-immobilized fiber

Supplementary Table S4. Patient characteristics before and after propensity score matching

	G-1 trial	JSEPTIC-DIC	SMD	G-1 trial	JSEPTIC-DIC	SMD
Patient number	75	3085		71	71	
Age, years	68.81 (11.34)	69.88 (14.36)	0.083	69.04 (11.36)	68.51 (11.00)	0.048
Sex, Male	41 (54.7)	1845 (59.8)	0.104	38 (54.0)	39 (54.9)	0.028
APACHE II	26.59 (4.43)	23.22 (8.67)	0.489	26.45 (4.48)	26.85 (3.99)	0.093
Total SOFA score	9.71 (3.11)	9.51 (3.93)	0.056	9.83 (3.12)	10.13 (3.17)	0.094
Past history						
Heart failure	7 (9.3)	175 (5.7)	0.139	7 (9.9)	5 (7.0)	0.101
Chronic kidney disease	10 (13.3)	254 (8.2)	0.165	10 (14.1)	11 (15.5)	0.04
Infection site			0.344			0.407
Urinary tract	21 (28.0)	505 (16.4)		20 (28.2)	12 (16.9)	
Lung	12 (16.0)	792 (25.7)		11 (15.5)	21 (29.6)	
Intra-abdominal	26 (34.7)	1004 (32.5)		26 (36.6)	22 (31.0)	
Other	12 (16.0)	572 (18.5)		10 (14.1)	12 (16.9)	
No data	4 (5.3)	212 (6.9)		4 (5.6)	4 (5.6)	
Intensive care treatment						
Mechanical ventilation	53 (70.7)	2209 (71.6)	0.021	52 (73.2)	49 (69.0)	0.093
Vasopressor medication	67 (89.3)	2413 (78.2)	0.305	63 (88.7)	67 (94.4)	0.204
Renal replacement therapy	61 (81.3)	1097 (35.6)	1.049	57 (80.3)	56 (78.9)	0.035
PMX	4 (5.3)	667 (21.6)	0.491	4 (5.6)	7 (9.9)	0.159
Low dose steroid medication	49 (65.3)	739 (24.0)	0.915	45 (63.4)	46 (64.8)	0.029
Laboratory results on the day of ICU admission						
Platelet counts ($\times 10^3/\mu L$)	145.17 (126.41)	143.35 (106.21)	0.016	145.31 (129.57)	158.29 (115.33)	0.106
PT-INR	1.51 (0.64)	1.55 (1.12)	0.05	1.52 (0.65)	1.43 (0.43)	0.168

FDP (μg/mL)	43.69 (57.84)	46.02 (108.29)	0.027	45.04 (58.92)	51.42 (103.37)	0.076
D-dimer (μg/mL)	17.78 (18.80)	23.84 (171.97)	0.05	17.92 (18.48)	22.53 (44.81)	0.134

Continuous variables are presented as means with standard deviation, and categorical variables are presented as numbers with percentages

SOFA, sequential organ failure assessment score; APACHE, acute physiologic assessment and chronic health evaluation; JSEPTIC-DIC, Japan Septic Disseminated Intravascular Coagulation; PMX, polymyxin B-immobilized fiber; ICU, intensive care unit; PT-INR, prothrombin time-international normalized ratio; FDP, Fibrin/fibrinogen degradation products

Supplementary Table S5. Patient characteristics after propensity score matching

	G-1 trial	FORECAST	SMD	G-1 trial	FORECAST	SMD
Patient number	75	909		72	72	
Age, years	68.81 (11.34)	70.30 (14.61)	0.114	68.86 (11.47)	67.24 (12.29)	0.137
Sex, Male	41 (54.7)	565 (62.2)	0.152	39 (54.2)	36 (50.0)	0.083
APACHE II	26.59 (4.43)	23.74 (8.84)	0.407	26.57 (4.52)	26.74 (4.20)	0.038
Total SOFA score	9.71 (3.11)	8.76 (3.73)	0.274	9.69 (3.14)	9.36 (3.05)	0.108
Past history						
Heart failure	7 (9.3)	143 (15.7)	0.194	7 (9.7)	8 (11.1)	0.045
Chronic kidney disease	10 (13.3)	66 (7.3)	0.201	9 (12.5)	5 (6.9)	0.188
Infection site			0.435			0.717
Urinary tract	21 (28.0)	158 (17.4)		21 (29.2)	12 (16.7)	
Lung	12 (16.0)	292 (32.1)		11 (15.3)	30 (41.7)	
Intra-abdominal	26 (34.7)	243 (26.7)		25 (34.7)	14 (19.4)	
Other	12 (16.0)	164 (18.0)		12 (16.7)	9 (12.5)	
No data	4 (5.3)	52 (5.7)		3 (4.2)	7 (9.7)	
Intensive care treatment						
Mechanical ventilation	53 (70.7)	406 (44.7)	0.545	50 (69.4)	50 (69.4)	<0.001
Vasopressor medication	67 (89.3)	640 (70.4)	0.486	64 (88.9)	65 (90.3)	0.045
Renal replacement therapy	61 (81.3)	293 (32.2)	1.141	58 (80.6)	54 (75.0)	0.134
PMX	4 (5.3)	81 (8.9)	0.139	4 (5.6)	3 (4.2)	0.065
Low dose steroid medication	49 (65.3)	289 (31.8)	0.712	46 (63.9)	45 (62.5)	0.029
Laboratory results on the day of ICU admission						
Platelet counts ($\times 10^3$/μL)	145.17 (126.41)	170.91 (150.48)	0.185	147.85 (127.90)	141.85 (113.91)	0.05
PT-INR	1.51 (0.64)	1.41 (0.78)	0.13	1.49 (0.62)	1.51 (1.10)	0.017
FDP (μg/mL)	43.69 (57.84)	43.73 (89.10)	0.001	43.41 (58.41)	66.48 (143.63)	0.21

D-dimer (μg/mL)	17.78 (18.80)	19.22 (50.78)	0.038	17.50 (18.91)	21.50 (43.47)	0.119

Continuous variables are presented as means with standard deviation, and categorical variables are presented as numbers with percentages

SOFA, sequential organ failure assessment score; APACHE, acute physiologic assessment and chronic health evaluation; FORECAST, Focused Outcomes Research in Emergency Care in Acute Respiratory Distress Syndrome, Sepsis, and Trauma; PMX, polymyxin B-immobilized fiber; ICU, intensive care unit; PT-INR, prothrombin time-international normalized ratio; FDP, Fibrin/fibrinogen degradation products

Supplementary Table S6. Patient characteristics before and after propensity score matching

	G-1 trial	JMDC	SMD	G-1 trial	JMDC	SMD
Patient number	75	1670		68	68	
Age, years	68.81 (11.34)	74.39 (14.10)	0.436	68.78 (11.52)	69.28 (14.01)	0.039
Sex, Male	41 (54.7)	1014 (60.7)	0.123	37 (54.4)	37 (54.4)	<0.001
SOFA subscores						
Liver function	0.71 (0.93)	0.44 (0.77)	0.315	1.76 (1.37)	1.74 (1.38)	0.156
Kidney function	1.72 (1.33)	1.24 (1.34)	0.359	0.62 (0.81)	0.75 (0.89)	0.021
Coagulation function	1.24 (1.18)	0.70 (0.97)	0.503	1.13 (1.18)	1.24 (1.11)	0.09
Past history						
Heart failure	7 (9.3)	595 (35.6)	0.664	7 (10.3)	8 (11.8)	0.047
Chronic kidney disease	10 (13.3)	309 (18.5)	0.142	10 (14.7)	10 (14.7)	<0.001
Infection site			1.01			0.809
Urinary tract	21 (28.0)	129 (7.7)		20 (29.4)	4 (5.9)	
Lung	12 (16.0)	433 (25.9)		12 (17.6)	12 (17.6)	
Intra-abdominal	26 (34.7)	411 (24.6)		22 (32.4)	22 (32.4)	
Other	12 (16.0)	117 (7.0)		10 (14.7)	13 (19.1)	
No data	4 (5.3)	580 (34.7)		4 (5.9)	17 (25.0)	
Intensive care treatment						
Mechanical ventilation	53 (70.7)	829 (49.6)	0.44	48 (70.6)	50 (73.5)	0.066
Vasopressor medication	67 (89.3)	1190 (71.3)	0.467	60 (88.2)	60 (88.2)	<0.001
Renal replacement therapy	61 (81.3)	333 (19.9)	1.556	54 (79.4)	56 (82.4)	0.075
PMX	4 (5.3)	22 (1.3)	0.225	4 (5.9)	3 (4.4)	0.067
Low dose steroid medication	49 (65.3)	513 (30.7)	0.739	42 (61.8)	40 (58.8)	0.06
Laboratory results on the day of ICU admission						
Platelet counts ($\times 10^3/\mu L$)	145.17 (126.41)	187.62 (109.25)	0.359	153.96 (129.41)	151.56	0.019

					(127.47)	
PT-INR	1.51 (0.64)	1.42 (0.83)	0.114	1.52 (0.66)	1.69 (1.04)	0.199
FDP (μg/mL)	43.69 (57.84)	41.11 (82.69)	0.036	44.30 (59.64)	47.99 (50.91)	0.066
D-dimer (μg/mL)	17.78 (18.80)	17.44 (35.82)	0.012	17.70 (19.16)	28.31 (38.02)	0.352

Continuous variables are presented as means with standard deviation, and categorical variables are presented as numbers with percentages

SOFA, sequential organ failure assessment score; JMDC, Japan Medical Data Center; PMX, polymyxin B-immobilized fiber; ICU, intensive care unit; PT-INR, prothrombin time-international normalized ratio; FDP, Fibrin/fibrinogen degradation products

Supplementary Table S7. Risk differences for primary outcomes after propensity-matching

Reference	G-1 trial	Control	RD	95% LCI	95% UCI	*P*-value
JSEPTIC-DIC	4 / 71 (5.6%)	16 / 71 (22.5%)	-0.17	-0.28	-0.06	0.003
FORECAST	4 / 72 (5.6%)	20 / 72 (27.8%)	-0.22	-0.34	-0.11	<0.001
JMDC	4 / 68 (5.9%)	26 / 68 (38.2%)	-0.32	-0.45	-0.20	<0.001

Categorical variables are presented as numbers with percentages
RD, Risk Difference; LCI, Low Confidence Interval; UCI, Upper Confidence Interval; JSEPTIC-DIC, Japan Septic Disseminated Intravascular Coagulation; FORECAST, Focused Outcomes Research in Emergency Care in Acute Respiratory Distress Syndrome, Sepsis, and Trauma; JMDC, Japan Medical Data Center

Supplementary Table S8. Adjusted risk ratios for primary outcomes after propensity score matching across three databases

Reference	Adjusted RR	95% LCI	95% UCI	*P*-value
JSEPTIC-DIC	0.25	0.09	0.72	0.011
FORECAST	0.17	0.06	0.47	<0.001
JMDC	0.20	0.07	0.55	0.002

RR, Risk ratio; LCI, Low Confidence Interval; UCI, Upper Confidence Interval; JSEPTIC-DIC, Japan Septic Disseminated Intravascular Coagulation; FORECAST, Focused Outcomes Research in Emergency Care in Acute Respiratory Distress Syndrome, Sepsis, and Trauma; JMDC, Japan Medical Data Center.
Covariates adjusted in the models were as follows:
JSEPTIC-DIC: infection site, heart failure, PMX use, vasopressor use, PT-INR, D-dimer, and platelet count;
FORECAST: infection site, chronic kidney disease, age, RRT use, total SOFA score, D-dimer, and FDP;
JMDC: infection site, SOFA liver function score, PT-INR, and D-dimer.

Supplementary Table S9. Adjusted risk differences for primary outcomes after propensity score matching across three databases

Reference	Adjusted RD	95% LCI	95% UCI	*P*-value
JSEPTIC-DIC	-0.16	-0.28	-0.04	0.009
FORECAST	-0.21	-0.34	-0.08	0.001
JMDC	-0.33	-0.53	-0.14	0.001

RD, Risk difference; LCI, Low Confidence Interval; UCI, Upper Confidence Interval; JSEPTIC-DIC, Japan Septic Disseminated Intravascular Coagulation; FORECAST, Focused Outcomes Research in Emergency Care in Acute Respiratory Distress Syndrome, Sepsis, and Trauma; JMDC, Japan Medical Data Center

Covariates adjusted in the models were as follows:

JSEPTIC-DIC: infection site, heart failure, PMX use, vasopressor use, PT-INR, D-dimer, and platelet count;

FORECAST: infection site, chronic kidney disease, age, RRT use, total SOFA score, D-dimer, and FDP;

JMDC: infection site, SOFA liver function score, PT-INR, and D-dimer.

Supplementary Table S10. Adjusted hazard ratio for primary outcomes after propensity score matching

Reference	Adjusted HR	95% LCI	95% UCI	*P*-value
JSEPTIC-DIC	0.23	0.07	0.77	0.017
FORECAST	0.2	0.06	0.71	0.013
JMDC	0.08	0.02	0.38	0.001

HR, Hazard Ratio; LCI, Low Confidence Interval; UCI, Upper Confidence Interval; JSEPTIC-DIC, Japan Septic Disseminated Intravascular Coagulation; FORECAST, Focused Outcomes Research in Emergency Care in Acute Respiratory Distress Syndrome, Sepsis, and Trauma; JMDC, Japan Medical Data Center
Covariates adjusted in the models were as follows:
JSEPTIC-DIC: infection site, heart failure, PMX use, vasopressor use, PT-INR, D-dimer, and platelet count;
FORECAST: infection site, chronic kidney disease, age, RRT use, total SOFA score, D-dimer, and FDP;
JMDC: infection site, SOFA liver function score, PT-INR, and D-dimer.

Supplementary Table S11. Hazard ratio for primary outcomes by IPTW

Reference	HR	95% LCI	95% UCI	*P*-value
JSEPTIC-DIC	0.195	0.057	0.668	0.009
FORECAST	0.191	0.06	0.607	0.005
JMDC	0.051	0.017	0.154	<0.001

HR, Hazard Ratio; LCI, Low Confidence Interval; UCI, Upper Confidence Interval; JSEPTIC-DIC, Japan Septic Disseminated Intravascular Coagulation; FORECAST, Focused Outcomes Research in Emergency Care in Acute Respiratory Distress Syndrome, Sepsis, and Trauma; JMDC, Japan Medical Data Center

Supplementary Table S12. Adjusted coefficient for secondary outcomes related to medical resource use after propensity score matching

Outcome<br>Reference	G-1 trial	Control	Adjusted OR	95% LCI	95% UCI	*P*-value
**ICU free periods (days)**						
JSEPTIC-DIC	19 [12, 21]	15 [0, 21]	2.07	1.07	3.97	0.030
FORECAST	20 [13, 21]	17 [8, 21]	3.10	1.51	6.35	0.002
JMDC	20 [11, 21]	15 [0, 22]	1.86	0.67	5.19	0.235
**Ventilator free periods (days)**						
JSEPTIC-DIC	24 [19, 28]	19 [0, 28]	2.30	1.17	4.56	0.016
FORECAST	25 [20, 28]	16 [0, 22]	9.51	4.55	19.9	<0.001
JMDC	24 [19, 28]	18 [1, 27]	5.55	2.04	15.15	0.001

Continuous variables are presented as median with IQR
OR, Odds ratio; LCI, Low Confidence Interval; UCI, Upper Confidence Interval; ICU, intensive care units; JSEPTIC-DIC, Japan Septic Disseminated Intravascular Coagulation; FORECAST, Focused Outcomes Research in Emergency Care in Acute Respiratory Distress Syndrome, Sepsis, and Trauma; JMDC, Japan Medical Data Center
Covariates adjusted in the models were as follows:
JSEPTIC-DIC: infection site, heart failure, PMX use, vasopressor use, PT-INR, D-dimer, and platelet count;
FORECAST: infection site, chronic kidney disease, age, RRT use, total SOFA score, D-dimer, and FDP;
JMDC: infection site, SOFA liver function score, PT-INR, and D-dimer.

Supplementary Table S13. Adjusted coefficient for secondary outcomes related to organ dysfunction after propensity score matching

Outcome<br>Reference	G-1 trial	Control	Coefficient	95% LCI	95% UCI	*P*-value
**ΔSOFA score**						
**ΔSOFA (day7 – day1)**						
JSEPTIC-DIC	-4.40 (3.88)	-1.83 (4.45)	-2.59	-4.184	-0.997	0.002
**ΔSOFA (day3 – day1)**						
JSEPTIC-DIC	-0.41 (4.09)	0.67 (3.66)	-1.191	-2.643	0.262	0.107
FORECAST	-0.59 (4.23)	1.11 (4.78)	-2.10	-3.591	-0.610	0.006
**ΔSOFA liver score (day7 – day1)**						
JMDC	-0.08 (0.85)	0.31 (1.10)	-0.631	-1.188	-0.074	0.027
**ΔSOFA kidney score (day7 – day1)**						
JMDC	-0.54 (1.52)	-0.40 (1.46)	-0.863	-1.928	0.202	0.111
**ΔSOFA coagulation score (day7 – day1)**						
JMDC	-0.14 (1.17)	0.43 (1.26)	-0.336	-1.076	0.404	0.369
**ΔSOFA liver score (day3 – day1)**						
JMDC	0.06 (0.93)	0.29 (0.85)	-0.49	-0.953	-0.027	0.038
**ΔSOFA kidney score (day3 – day1)**						
JMDC	0.35 (1.67)	0.16 (1.13)	-0.017	-0.815	0.78	0.966
**ΔSOFA coagulation score (day3 – day1)**						
JMDC	0.86(1.07)	0.73 (0.80)	0.027	-0.526	0.58	0.923
**ΔDIC score**						
**ΔDIC score (day7 – day1)**						

JSEPTIC-DIC	-1.47 (2.40)	-1.25 (2.30)	-0.139	-1.041	0.764	0.761
JMDC	-1.4 (2.37)	0.71 (2.62)	-1.59	-3.41	0.227	0.085
**ΔDIC score (day3 – day1)**						
JSEPTIC-DIC	-0.16 (2.10)	-0.30 (2.12)	0.292	-0.509	1.093	0.472
FORECAST	-0.30 (2.01)	-0.07 (2.33)	-0.420	-1.248	0.408	0.317
JMDC	-0.14 (2.07)	0.83 (2.41)	-0.842	-2.23	0.547	0.231

Continuous variables are presented as means with standard deviation

LCI, Low Confidence Interval; UCI, Upper Confidence Interval; SOFA, Sequential Organ Failure Assessment; DIC, disseminated intravascular coagulation; JSEPTIC-DIC, Japan Septic Disseminated Intravascular Coagulation; FORECAST, Focused Outcomes Research in Emergency Care in Acute Respiratory Distress Syndrome, Sepsis, and Trauma; JMDC, Japan Medical Data Center

Covariates adjusted in the models were as follows:

JSEPTIC-DIC: infection site, heart failure, PMX use, vasopressor use, PT-INR, D-dimer, and platelet count;

FORECAST: infection site, chronic kidney disease, age, RRT use, total SOFA score, D-dimer, and FDP;

JMDC: infection site, SOFA liver function score, PT-INR, and D-dimer.

Supplementary Figure. 1 Absolute standardized mean difference plot before and after propensity score matching. (A) G-1 vs. JSEPTIC-DIC. (B) G-1 vs. FORECAST. (C) G-1 vs. JMDC. JSEPTIC-DIC, Japan Septic Disseminated Intravascular Coagulation; FORECAST, Focused Outcomes Research in Emergency Care in Acute Respiratory Distress Syndrome, Sepsis, and Trauma; JMDC, Japan Medical Data Center; SOFA, Sequential Organ Failure Assessment; APACHE, Acute Physiologic Assessment and Chronic Health Evaluation; CKD, chronic kidney disease; PMX, polymyxin B-immobilized fiber; MV, mechanical ventilation; RRT, renal replacement therapy; PT-INR, prothrombin time-international normalized ratio; FDP, Fibrin/fibrinogen degradation products.

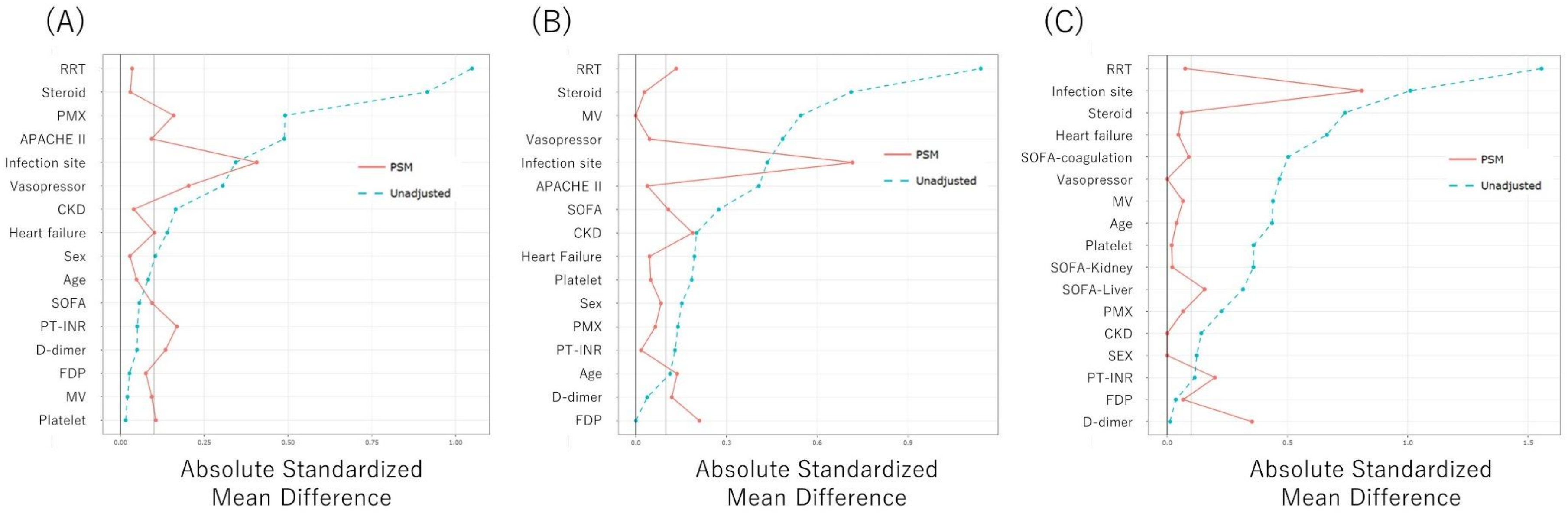

Supplement: Supplementary file 1 — Supplementary Material 1. [file 40560_2026_872_MOESM1_ESM.pdf]
